# Simultaneously quantifying *K*_1_, *k*_2_, and blood volume in myocardial imaging with overlapping volumes of interest: practical identifiability

**DOI:** 10.1088/1361-6560/ae62c5

**Published:** 2026-10-09

**Authors:** Dale J Stentz, Marie A Guerraty, Scott D Metzler

**Affiliations:** 1 Department of Radiology, University of Pennsylvania, Philadelphia, PA, United States of America; 2 Division of Cardiovascular Medicine, Department of Medicine, University of Pennsylvania, Philadelphia, PA, United States of America

**Keywords:** dynamic imaging, myocardial blood flow, structural identifiability, practical identifiability

## Abstract

*Objective.* Dynamic imaging in emission computed tomography allows the determination of physiological parameters that have important clinical implications. In myocardial imaging, three parameters of interest are the uptake and washout rates, ${K_1}$ and ${k_2}$, respectively, and the blood-volume fraction, V. Theoretically, these parameters can be determined from time-activity curves (TACs) of the blood pool and the myocardium from injection and uptake of a perfusion tracer, if the matrix describing the relationship between the true TACs and the observed TACs is known. Herein, we numerically confirm these theoretical predictions and study the impact of experimental noise and matrix element uncertainty on these three parameters. *Approach.* Using a one-compartment model, we simulated TACs for different values of ${K_1}$, ${k_2}$, and V. A mixing matrix involving recovery coefficients and spillover fractions was used to describe the transformation of the true concentration to the observed concentrations. Kinetic model fitting was employed to measure the three physical parameters, and we measured the bias of these fit results with respect to the true simulated values. We investigated a range of mixing matrices, rates of uptake and washout, and blood volume fractions. *Main results.* We confirmed the theoretical prediction that ${K_1}$, ${k_2}$, and V can be simultaneously measured accurately if and only if all elements of the mixing matrix are known. There was an approximately linear effect of uncertainty in the measurement of the three physical parameters to the noise level on the TACs. Consistent with previous results, greater mixing via the mixing matrix or the blood-volume fraction resulted in greater uncertainty. *Significance.* This work shows we can accurately measure the physical parameters like ${K_1}$ while also quantifying the uncertainty in their measurement due to noise on the TACs and mixing-matrix uncertainty.

## 1. Introduction

The measurement of myocardial blood flow (MBF) is an important diagnostic tool for heart diseases (e.g. coronary microvascular disease) (Camici and Crea 2007, Guerraty *et al* 2020, Del Buono *et al* 2021, Schindler *et al* 2023, Scarica *et al* 2025). A time-dependent reconstructed image can be acquired using an emission computed tomography scanner capable of dynamic imaging. In the case of MBF, this requires the measurement of the activity concentration of the tracer injection into the blood (i.e., input function) and the uptake into the myocardium tissue. Collectively, these activity concentration measurements with respect to time are hereafter referred to as time activity curves (TACs).

We consider the one-compartment model (Metzler *et al* 2026), governed by

\begin{equation*}\frac{{{\mathrm{d}}{C_T}}}{{{\mathrm{d}}t}} = {K_1}{C_A} - {k_2}{C_T},\end{equation*}

where ${C_T}$ and ${C_A}$ are the activity concentrations for the myocardial tissue (T) and arterial blood (A), respectively, defined via their respective volume of interests (VOIs). The tracer uptake constant for the tissue is denoted as ${K_1}$ while the washout (tissue to blood) is ${k_2}$. Herein, we explicitly consider ${k_2}$ > 0 in contrast to previous work (Johnson *et al* 2023) where ${k_2}$ = 0. The measurement of ${K_1}$ allows for the determination of the MBF using the extraction fraction (Klein *et al* 2010, Guerraty *et al* 2021). Experimentally, we measure two observed concentrations: ${C_{{\mathrm{Myo}}}}$ for the myocardial VOI and ${C_{{\mathrm{BP}}}}$ for the VOI of the blood pool. The relation between the observed and true concentrations (${C_T}$ and ${C_A}$) is given by equation (2) (Johnson *et al* 2023, Metzler *et al* 2026),

\begin{equation*}\begin{array}{*{20}{c}} {\left( {\begin{array}{*{20}{c}} {{C_{\mathrm{Myo}}}} \\ {{C_{\mathrm{BP}}}} \end{array}} \right) = \left( {\begin{array}{*{20}{c}} {{R_T}}&amp;{{F_A}} \\ {{F_T}}&amp;{{R_A}} \end{array}} \right)\left( {\begin{array}{*{20}{c}} {\left( {1 - V} \right){C_T} + V{C_A}} \\ {{C_A}} \end{array}} \right){\text{ }}} \end{array}.\end{equation*}

The blood-volume fraction, V, represents the fraction of arterial concentration in the myocardial tissue that is observed in the myocardium (i.e., the fraction of blood in the myocardial VOI). The four parameters {${R_T}$, ${R_A}$, ${F_T}$, ${F_A}$ } taken together are the mixing matrix. The diagonal elements, ${R_T}$ and ${R_A}$, are like recovery coefficients (Cherry *et al*); they act as a scaling for the overall quantification between the true and observed concentration. They can be estimated from phantom studies (Johnson *et al* 2019) or via direct measurement (Cherry *et al*, Guerraty *et al* 2021) where the true activity concentration is known or measured. The off-diagonal elements, ${F_T}$ and ${F_A}$, can be considered as spillover fractions (Hutchins *et al* 1992, Klein *et al* 2010, Ocneanu *et al* 2017) where the true concentration of one VOI mixes with the other (e.g. ${F_A}{C_A}$ contaminates the measurement of ${C_{{\mathrm{Myo}}}}$). Using the same radioisotope but with different tracer kinetics (Guerraty *et al* 2021) allows for the measurement ${F_T}$ given ${R_T}$ (i.e. when ${k_2}$ = 0) as well as ${F_A}$ given ${R_A}$ (i.e. when ${K_1}$ = 0 via labeled red-blood cells for example). Phantom studies (Johnson *et al* 2019) can also be employed to estimate ${F_T}$ and ${F_A}$. Contrast-enhanced CT provides another option to directly measure the spillover-volume fractions (Mohy-Ud-Din *et al* 2018, Yousefi *et al* 2023). Unlike the blood-volume fraction, which represents a physiological parameter, the mixing-matrix elements are indicative of the resolution performance of the scanner and the nature of the drawn VOIs.

The kinetic model described in equations (1) and (2) provides a way (e.g. through a fitting algorithm such as least-squares or maximum likelihood, etc) to extract the physical parameters of interest: ${K_1}$, ${k_2}$, and V. All three parameters are potentially impactful for kinetic analysis (e.g. in estimating the MBF via ${K_1}$, etc). However, it is possible that model might not provide a unique solution for the parameters of interest: the same observed TACs could be produced with different parameters. Thus, we found theoretically in the corresponding companion paper (Metzler *et al* 2026) that the four parameters of the mixing matrix must be known for a unique solution for ${K_1}$, $k_2$, and V. Conversely, it may be possible to extract some of the harder-to-estimate mixing-matrix parameters (i.e. ${F_T}$ and/or ${F_A}$) and still achieve a unique set of fitting parameters if some combination of ${K_1}$, ${k_2}$, and V is known; for example, if a tracer is known not to washout (i.e. $k_2$ = 0) (Metzler *et al* 2026).

We define *structural identifiability* (Raue *et al* 2009, Chis *et al* 2011, Guillaume *et al* 2019, Wieland *et al* 2021) as the ability to identify all model parameters when the data are noiseless and exact, as considered in (Metzler *et al* 2026). Thus, a *structurally non-identifiable* model can have the same observable output (e.g. experimental TACs) for different model parameters. This is in contrast with *practical identifiability* (Raue *et al* 2009, Gábor *et al* 2017, Zuo *et al* 2019, Wieland *et al* 2021), which evaluates the impact on the estimation of model parameters via the impact of noise on the observable TACs (${C_{{\mathrm{Myo}}}}$ and ${C_{{\mathrm{BP}}}}$). The concern is that a model may be structurally identifiable, but some parameters may be practically non-identifiable (e.g. a large or infinite confidence interval) for a given tolerance of the observable data to the estimated kinetic model.

Here, we simulate TACs with different noise characteristics and parameter values to generate the observable TACs, including noise. We fit the TACs to estimate the physiological parameters using a least-squares fitting algorithm for different scenarios. We then compare the work herein with the analytical treatment of the same problem (Metzler *et al* 2026) without noise to confirm the cases where a unique solution can be achieved and also determine the expected noise on the physiological parameters as a function of data noise for the different scenarios. In this regard, this work represents an expansion of (Johnson *et al* 2023) and, beyond the confirmation of the theoretical prediction of (Metzler *et al* 2026), provides a way to estimate the uncertainty of $K_1$, $k_2$, and V given the noise on the underlining TAC or uncertainty in the mixing-matrix elements.

## 2. Methods

### 2.1. Model considerations

We consider the one-compartment model defined in equations (1) and (2) where ${k_2}$ > 0. This reversible one-compartment kinetic model reflects the use of a tracer like ^99m^Tc-Teboroxime in contrast to previous work with ^99m^Tc-Sestamibi where ${k_2}$ = 0 (Johnson *et al* 2019, 2023, Guerraty *et al* 2021). Further, our recent studies with ^99m^Tc-Teboroxime show it has a higher extraction fraction (≈90% versus 65% first pass extraction over MBF range of 0–4.5 ml min^−1^mg^−1^), making it better suited for the elevated MBFs seen in mice, but with significant redistribution relative to Sestamibi (Boschi *et al* 2022).

### 2.2. TAC simulations

The true activity concentrations, ${C_T}$ and ${C_A}$, are derived from an impulse function, which emulates the bolus injection into the arterial volume. The details of this simulation procedure are given in the supplementary material. The only kinetic model parameters used for this true concentration generation are ${K_1}$ and ${k_2}$ in accordance with equation (1). We generate simulation data for ${K_1}$ = {0.5, 1.5, 2.5} ml min^−1^g^−1^ and ${k_2}$ between 0.01 and 0.10 of ${K_1}$. For simplicity, we represent the units of $K_1$ using min^−1^ for all relevant figures. The arterial concentration visually matched the peak region (i.e. approximately the same peak width for ${K_1}$ = 1.5 ml min^−1^g^−1^) of the gamma variant input function used in (Johnson *et al* 2023).

These effectively continuous true activity concentrations spanned 300 s and match the typical time duration used in our experiments with mice and previous simulations (Johnson *et al* 2019, 2023, Guerraty *et al* 2021). For most of the results presented here the continuous concentration functions were converted to ${N_{\mathrm{f}}} = 30$ time frames, each representing the average over a 10 s frame. The 10 s frame-length provides a good tradeoff in terms of sensitivity and signal-to-noise to time resolution and matches the nominal time-frame binning used in Johnson *et al* (2023). The simulation data are then converted to the observed concentrations, *$C_{{\mathrm{Myo}}}$* and ${C_{{\mathrm{BP}}}}$, using equation (2) and the remaining 5 parameters: the blood-volume fraction and the mixing matrix. For the blood-volume fraction we consider V = {0, 0.05, 0.10, 0.20, 0.30}. For the mixing-matrix parameters, we consider four cases (denoted as mixing matrices M1, M2, M3, and M4), which are shown in table 1.

**Table 1.** Mixing matrices: M1–M4.

Matrix #	${R_T}$	*R_A_*	*F_T_*	${F_A}$
M1	1.00	1.00	0.00	0.00
M2	0.90	0.95	0.10	0.05
M3	0.80	0.85	0.20	0.15
M4	0.70	0.75	0.30	0.25

### 2.3. Phase and noise realizations

From the simulation of section 2.2, we generate different realizations via two sources of randomness. First, we introduce a random time shift within one time frame (between 0 and 10 s) to the data prior to binning, to account for the experimentally unknown alignment of the data binning and the tracer injection’s appearance in the FOV. We hereafter refer to different time shifts as phases of the same underlying simulation parameters. Second, we introduce Gaussian noise, which is applied independently to each observed concentration for each time frame for each phase. We require activity concentrations to be non-negative; the concentration is set to 0 if noise would give a negative concentration. The noise level varies between 0% (i.e. noiseless) to 10% in 1% increments. Like (Johnson *et al* 2023), these noise realizations are intended to represent any aleatoric variation in our TACs.

### 2.4. Kinetic parameter fitting

Our estimation of model parameters comes from our in-house kinetic fitting software, which finds the parameter values through minimization of a fit metric in equation (3) that we designate herein as ${\tilde \chi^2}$, where $\langle {C_{ij}}\rangle $ is the fit model concentrations (based on fit parameters) and ${C_{ij}}$, the observed activity concentration at time frame i for each observed VOI *j*

\begin{equation*} {{\tilde \chi }^2} = \mathop \sum \limits_{j \in \left\{ {{\mathrm{Myo}},{\mathrm{BP}}} \right\}} \mathop \sum \limits_{i = 1}^{{N_{\mathrm{f}}}} \frac{{{{\left( {\left\langle {{C_{ij}}} \right\rangle - {C_{ij}}} \right)}^2}}}{{{{\Delta }}_{ij}^2}} .\end{equation*}

Our results focus on using a weighting (or error term) ${{{\Delta }}_{ij}}$ of unity for all time frames, corresponding to a least-squares fit. However, we also consider a Poisson uncertainty weighting (${{{\Delta }}_{ij}} = \sqrt {{C_{ij}}} $) as well as a Gaussian uncertainty weighting (${\Delta}_{ij}$ is equal to ${C_{ij}}$ times the Gaussian noise level). The last weighting model explicitly matches our noise generation of section 2.3 and allows for the fitting metric ${\tilde \chi^2}$ to be interpreted as properly chi-squared (${\chi ^2}$) distributed. We note that our simulations produce ${C_{ij}}$ > 0. Additionally, the noiseless (0% noise) case is undefined for the Gaussian uncertainty weighting and is not included in our results.

We utilize a modified Akima spline (Akima 1970, 1974) to allow for finer sampling of time points (0.25 s in comparison to 10 s time-frame) of the true concentrations. This amounts to the introduction of a knot parameter representing the arterial concentration at each time frame. These knots (${N_{\mathrm{f}}}$ = 30 in total) are free parameters in the fit. Additionally, three static knots set at zero concentration are generated at t={−20, −10, 0} seconds, ensuring the spline is physical (e.g. non-negative concentrations) as well as satisfying the initial conditions (i.e. ${C_A}\left( 0 \right) = 0$). The resulting Akima spline represents the fit-model estimation of the arterial concentration $\left( {{C_A}} \right)$ with this finer time binning. Following the procedure of Johnson *et al* (2019, 2023), numerical integration of equation (1) then gives the tissue concentration (${C_T}$) of the fit model. The observed concentration for the fit model at each time frame, $\left\langle {{C_{ij}}} \right\rangle $, is calculated by taking the average value over the time frame duration (matching the procedure of section 2.2) and applying the mixing governed by equation (2).

### 2.5. Tests for parameter identifiability

#### 2.5.1. Structural identifiability

Using the noiseless simulated TACs and the full range of mixing matrices and physical parameter values (see section 2.2), we investigate the absolute bias or the relative error in our fit parameters relative to the true simulation values. Additionally, we consider three scenarios based on which mixing-matrix elements are included as fit parameters: only ${F_T}$, only ${F_A}$, and none included. These correspond to Trials #3–5 of (Johnson *et al* 2023), respectively, but without the constraint of ${k_2} = $ 0. For fixed (non-fit) parameters of the mixing matrix, the values used are always accurate to the simulation value.

To test for structural non-identifiability (Chis *et al* 2011, Guillaume *et al* 2019, Wieland *et al* 2021) and confirm the predictions of (Metzler *et al* 2026), we fit with ${F_T}$ systematically biased across a range of 41 values (including end points) and plot the fitting metric (${\tilde \chi ^2}$) in addition to bias and relative error plots of the three physical free fit variables versus the fixed ${F_T}$ value. This is then repeated with ${F_A}$ systematically biased instead. The range of the systematically biased ${F_T}$ (‘Forced ${F_T}$ bias’) and ${F_A}$ (‘Forced ${F_A}$ bias’) is constrained to the physically allowable range given by the predictions of Metzler *et al* (2026) to maintain the fit invariant.

#### 2.5.2. Fitting parameter uncertainty due to noise

We investigate the effect of our Gaussian-noise and phase-shift realizations (section 2.3) when fitting with all elements of the mixing matrix accurately known and passed as fixed parameters. We calculate the relative error of ${K_1}$ and ${k_2}$ as well as the absolute bias of *V* of each replicate and compose the mean and standard deviation across the 100-noise realization. We perform this analysis for all three different weighting models in our fitting (section 2.4).

#### 2.5.3. Error-propagation estimation

The procedure of section 2.5.1 assumes that the fixed mixing-matrix elements are accurately and precisely known (i.e. the fit uses the true simulated values with no uncertainty). We investigate the propagation effect on the fitted value of ${K_1}$, ${k_2}$, and V due to there being an uncertainty on the mixing-matrix elements. We denote the absolute uncertainty for a given mixing-matrix element as $\sigma \left( {{M_i}} \right)$ where ${M_i} \in \left\{ {{R_T},{\text{ }}{R_A},{F_T},{F_A}} \right\}$.

We developed a Monte Carlo (MC) simulation that randomly generates a Gaussian distribution centered on the true simulation value of the mixing-matrix element with standard deviation equal to the assigned relative uncertainty of the same. To distinguish the true simulated value from the randomly generated ones we employ the ‘primed’ notation of (Johnson *et al* 2023, Metzler *et al* 2026) (e.g. $R_T^{\prime}$ is a sample from the normal distribution with mean *R_T_* and standard deviation ${\sigma(R_T)}$). We investigate uncertainties between 0.1% and 5.0% of the mixing-matrix element in 0.1% increments. This generation is repeated for all mixing-matrix elements. If a generated value is unphysical (e.g. $F_T^{\prime} &lt; 0$), it is rejected, and a new value is generated. Using the true simulation parameters and the four generated mixing-matrix elements, we obtain $K_1^{\prime}$, $k_2^{\prime}$, and $V^{\prime}$ (see equations (4)–(6)) derived from the theoretical prediction of Metzler *et al* (2026). If any of the derived physical parameters are unphysical (e.g. $k_2^{\prime} &lt; 0$) the event is vetoed, and a new random generation of matrix elements begins. We generate a total of 1 million events (10^6^) for each parameter space.

In equations (4)–(6), the ‘unprimed’ variables (e.g. V) refer to the true parameter value whereas the primed (e.g. $V^{\prime}$) are the values that produce an equivalent observed TAC (Metzler *et al* 2026)

\begin{equation*}{K_1}^{\prime} = \frac{X}{{X^{\prime}}}{K_1}\end{equation*}

\begin{equation*} k_2^{\prime} = {k_2} - \left( {\frac{{{K_1}\left( {1 - V} \right)}}{{\left( {{R_T}V + {F_A}} \right)\left( {F_T^{\prime} V^{\prime} + R_A^{\prime}} \right)}}} \right)\frac{Y}{{X^{\prime}}}\end{equation*}

\begin{equation*} V^{\prime} = \frac{{\left( {R_A^{\prime}{F_A} - {R_A}F_A^{\prime}} \right) + \left( {{R_T}R_A^{\prime} - {F_T}F_A^{\prime}} \right)V}}{{\left( {R_T^\prime{R_A} - F_T^\prime{F_A}} \right) + \left( {R_T^\prime{F_T} - {R_T}F_T^{\prime}} \right)V}} .\end{equation*}

When all the mixing-matrix elements are accurately known the physical parameters collapse to the true value (e.g. $V^{\prime} = V$). Equations (5) and (6) utilize convenience variables X, $X^{\prime}$, and Y, which are given in equations (7)–(9)

\begin{equation*} X = \left( {\frac{{{R_T}}}{{{R_T}V + {F_A}}} - \frac{{{F_T}}}{{{F_T}V + {R_A}}}} \right)\left( {1 - V} \right) \end{equation*}

\begin{equation*} X^{\prime} = \left( {\frac{{R_T^{\prime}}}{{R_T^{\prime} V^{\prime} + F_A^{\prime}}} - \frac{{F_T^{\prime}}}{{F_T^{\prime} V^{\prime} + R_A^{\prime}}}} \right)\left( {1 - V^{\prime}} \right) \end{equation*}

\begin{equation*} Y = \left( {{R_T}F_T^{\prime} - R_T^\prime{F_T}} \right)\left( {1 - V^{\prime}} \right).\end{equation*}

From our resulting MC distributions, we compute the sample mean and standard deviation,, for each physical parameter, $P \in \{ {K_1}$, ${k_2}$, V}. We define the relative uncertainty as the ratio of standard deviation of the parameter to its true value (${s_P}/P$) calculated as a percentage. Hereafter, we will use this relative error like metric to categorize the uncertainty of the physical parameters to meaningfully compare results between each other (e.g. different ${K_1}$ values, ${K_1}$ compared to, $k_2$ etc).

In addition to the MC generation procedure, we also estimate the error propagation directly by analytical approximation. For a physical variable $P^{\prime} \in \{ K_1^{\prime}$, $k_2^{\prime}$, $V^{\prime}\} $ defined in equations (4)–(6) the variance of the corresponding P can be estimated, to first order, as

\begin{equation*}\begin{array}{*{20}{c}} {\sigma _P^2 \cong \mathop {\mathop \sum \nolimits }\limits_{\forall {\text{ }}i} {{\left( {\frac{{\partial P^{\prime}}}{{\partial M_i^{\prime}}}{|_M}} \right)}^2}\sigma _i^2} \end{array}\end{equation*}

where $M_i^{\prime} \in \left\{ {R_T^{\prime},{\text{ }}R_A^{\prime},F_T^{\prime},F_A^{\prime}} \right\}$ and ${\sigma _i} = \sigma \left( {{M_i}} \right)$ is the absolute uncertainty for a given mixing-matrix element. The appendix gives the analytical form for the uncertainty of each physical variable using equation (10). The partial derivatives are evaluated at the true simulated values for the mixing matrix ($M^{\prime}$ = M). Equation (10) assumes the correlation coefficient of non-identical mixing-matrix elements is 0; this assumption will be checked using the MC results. These analytical approximation results are compared to the MC results using the same relative uncertainty calculation but defined as ${\sigma _P}/P$ (as a percentage).

## 3. Results

Figure 1 shows an example simulation of TACs with 0% and 3% noise along with the corresponding kinetic fit using the same physical and mixing parameters. The fit describes the simulated data well in both cases. The examples shown are for using a least-squares fit (${{{\Delta }}_{ij}} = 1$) with the M3 mixing matrix (see table 1) and blood-volume fraction V of 0.1. Variations between the fit parameters (e.g. ${K_1}$) of different weighting models (e.g. Gaussian uncertainty weighting) are more than an order of magnitude smaller than the variance between realizations.

**Figure 1.**
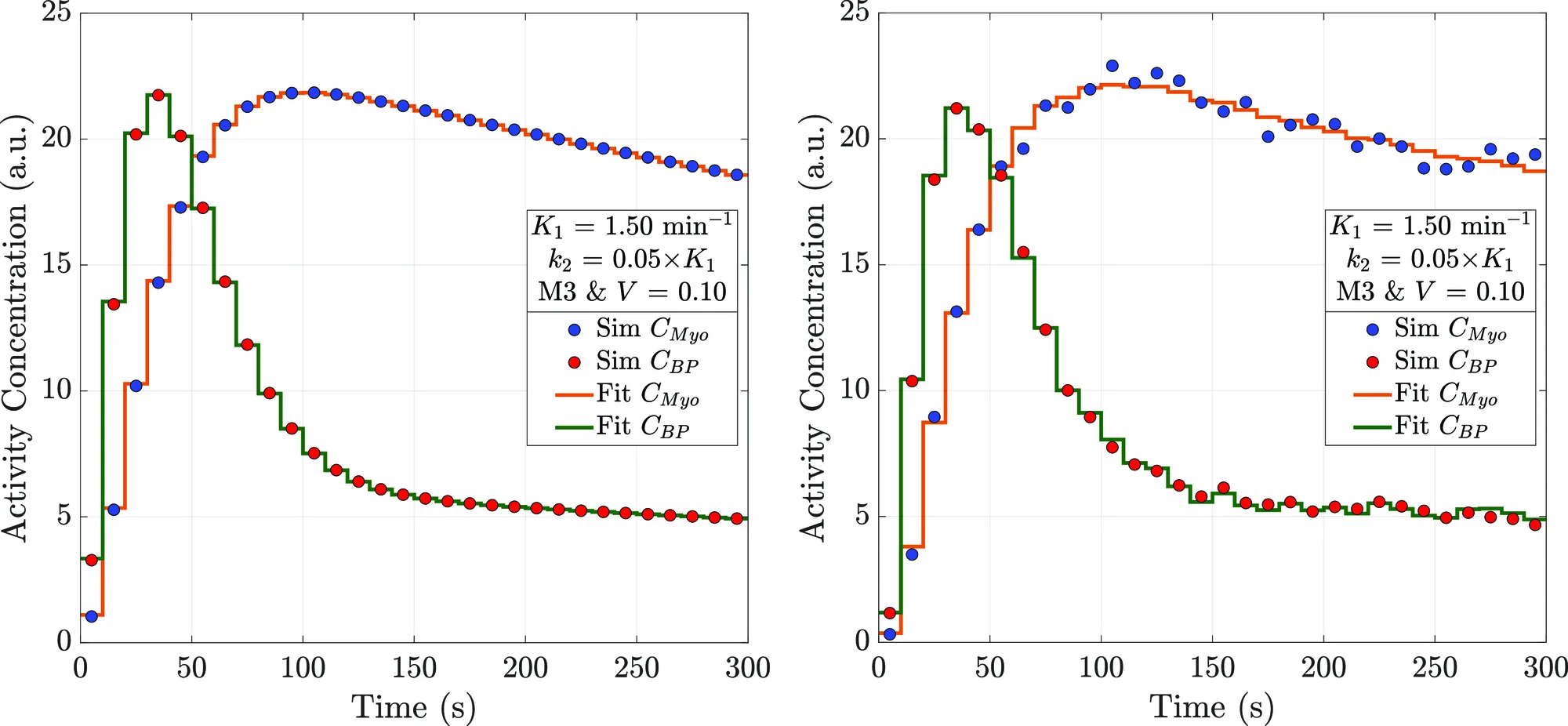
Simulated TACs (circle markers) and the kinetic fit (solid line) for no noise (left) and 3% Gaussian noise (right). As annotated in the legend, these examples are for ${K_1}$ = 1.5 ml min^−1^g^−1^, $k_2/K_1$ = 0.05, and V = 0.1.

### 3.1. Structural identifiability

#### ${F_A}$ Free parameter (forced ${F_A}$ bias)

3.1.1.

For a given set of kinetic and mixing parameters, the fit metric was uniform (flat) for our noiseless simulation results across the prescribed forced $F_A$ bias range. For example, in our default least squares metric, the variation was less than one part in a million. Therefore, the resulting fits to the observed TACs were indistinguishable across the range of ${F_A}$ values. However, the returned fit parameters $K_1$ and V were biased depending on the forced ${F_A}$ bias as shown in figure 2. Each figure includes the four different mixing matrices for the physical parameters ${K_1}$ = 1.5 ml min^−1^g^−1^, ${k_2}/{K_1}$ = 0.05, and V = 0.1 as well as the prediction error via (Metzler *et al* 2026). The colored markers of figure 2 are the average relative error while the error bars are the standard deviation across 10 phases for $K_1$ and *V*, respectively. Both physical variables were inversely dependent. While the relative error in V was observed to be linear, the $K_1$ relative error was distinctly nonlinear with greater mixing (e.g. M4 compared to M1). In this example we observe a ±0.05 change in ${F_A}$ from the true value can generate a ≈10% error in ${K_1}$ and ≈75% in V for mixing matrix 3 (M3). The behavior of figure 2 was replicated across our simulated physical-parameter space and also confirms the previous results of Johnson *et al* (2023) when ${k_2}$ = 0. We conclude that ${K_1}$ and V are not identifiable, however, for ${k_2}$ (not shown) the results are independent of the set ${F_A}$ value and accurate to the true value ($ \approx \pm $ 0.1% bias).

**Figure 2.**
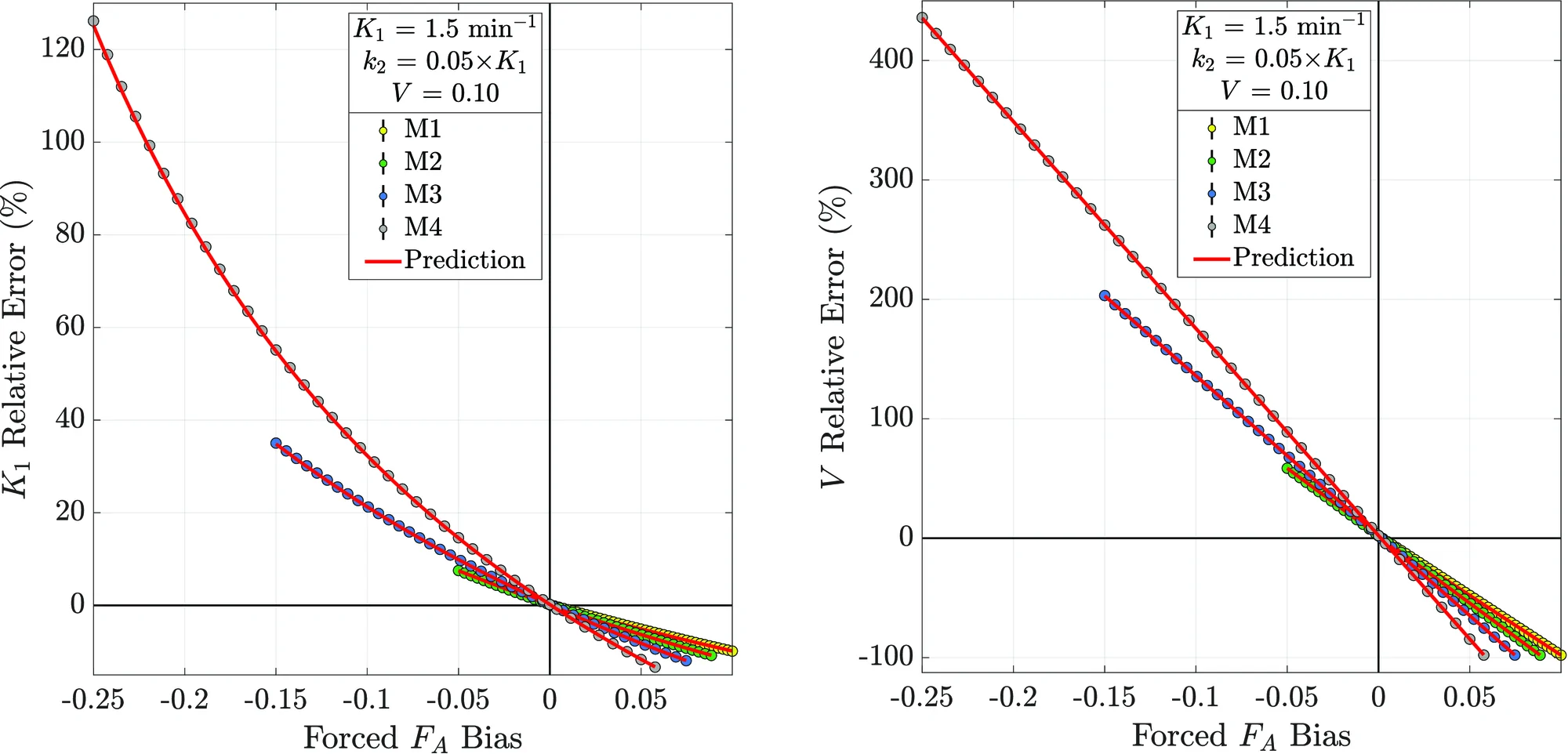
Relative error for ${K_1}$ (left) and V (right) versus the force ${F_A}$ bias for all four mixing matrices. Each marker (error bars) represents an average (standard deviation) of 10 unique fits of 10 phases while the red lines are the theoretical prediction from Metzler *et al* (2026). As annotated in the legend, these examples are for ${K_1}$ = 1.5 ml min^−1^g^−1^, ${k_2}/{K_1}$ = 0.05, and V = 0.1. On both plots the error bars are not observable due to the large relative error ranges.

#### ${F_T}$ Free parameter (forced ${F_T}$ bias)

3.1.2.

The least-squares fit metric was observed to be correlated with forced ${F_T}$ bias (i.e. not uniform and flat as observed in section 3.1.1). This dependence was observed only at high precision in the fit metric. By comparison, the deviation of the fit metric due to the 10 different time phases was over 100 times larger. In this context, the observed TACs were identical across the ${F_T}$ bias range. Figure 3 shows the relative error of ${K_1}$, $k_2$, and V*V* versus forced $F_T$ bias using the same physical parameters, etc as figure 2. Like section 3.1.1, the predicted error of Metzler *et al* (2026) is in excellent agreement with the simulated results. However unlike in the forced ${F_A}$ bias case, ${k_2}$ shows a dependence with forced ${F_T}$ bias as predicted. In all cases the dependence was approximately linear with only ${k_2}$ (negatively) correlated with forced $F_T$ bias. We observe, ${K_1}$ was the least sensitive to ${F_T}$ bias, while ${k_2}$ was the most sensitive with over 100% error for a 0.05 bias in $F_T$. These trends highlighted in figure 3 hold across the larger simulated physical-parameter space we measured. We conclude that all three physical variables are not identifiable, and this is consistent with the prediction of Metzler *et al* (2026).

**Figure 3.**
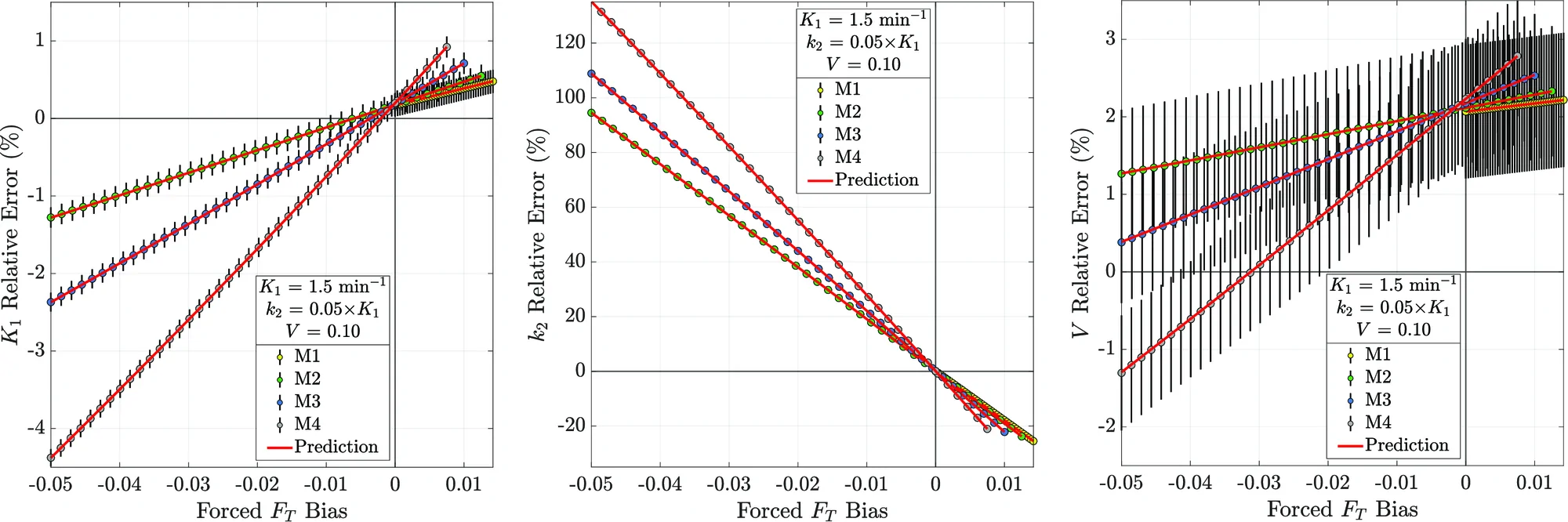
Relative error for ${K_1}$ (left), ${k_2}$ (middle), and V (right) versus the force *F_T_* bias for all four mixing matrices. Each marker (error bars) represents an average (standard deviation) of 10 unique fits of 10 phases while the red lines are the theoretical prediction from Metzler *et al* (2026). As annotated in the legend, these examples are for ${K_1}$ = 1.5 ml min^−1^g^−1^, ${k_2}/{K_1}$ = 0.05, and V = 0.1.

### 3.2. Fitting parameter uncertainty due to noise

As prescribed by section 2.5.2, we investigated the average and standard deviation of all three physical parameters (${K_1}$, ${k_2}$, and V) in conjunction with Gaussian noise across 100 noise replicates. The results use the true mixing matrix as fixed parameters passed to the fit. Figure 4 shows the relative error (or absolute bias in the case of V) versus the Gaussian noise (as a percentage) for the case where ${K_1}$ = 1.5 ml min^−1^g^−1^ and ${k_2}/{K_1}$ = 0.05. We highlight three mixing cases {M1 & V = 0.0, M3 & V = 0.1, M4 & V = 0.2}, which span from no mixing to maximal mixing. These results use the least squares as the fit metric (${\tilde \chi ^2}$ with ${\Delta}_{ij}$ *=*1). The error bars in figure 4 represent the standard deviation of the 100 noise replicates. The small horizontal offset at each percentage of noise is for clarity, to separate the three mixing cases for the reader.

**Figure 4.**
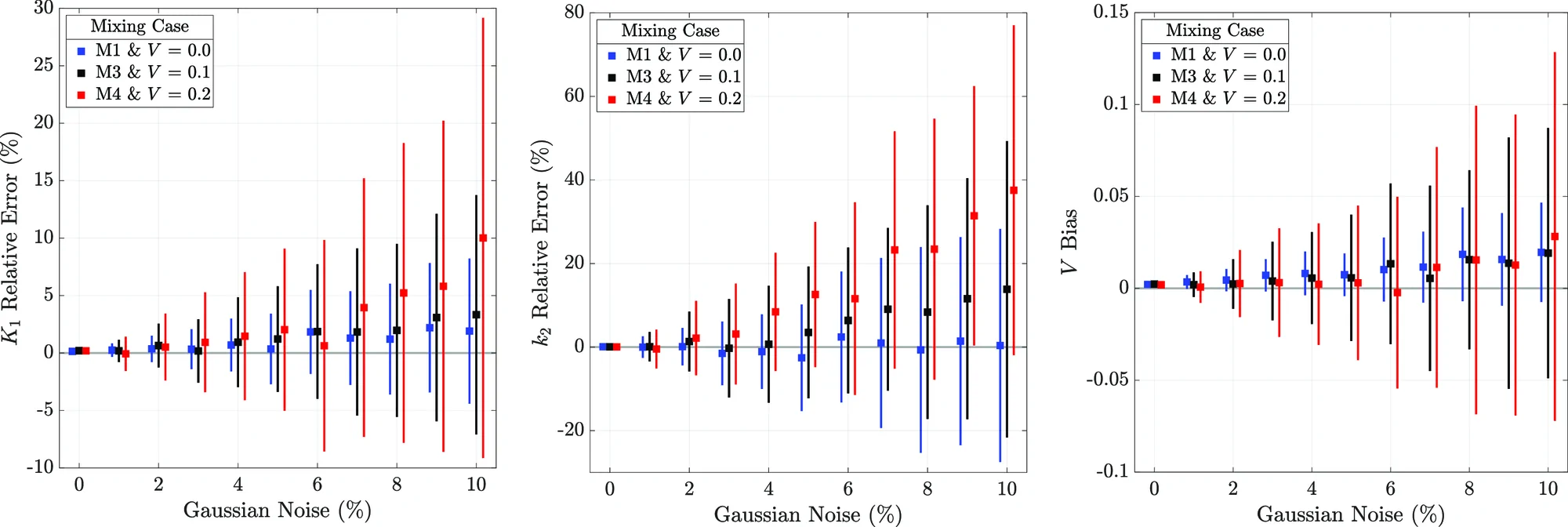
The relative error for ${K_1}$ (left) and $k_2$ (middle) as well as the absolute bias for V (right) versus the Gaussian noise percentage on the observed TACs. These results use ${K_1}$ = 1.5 ml min^−1^g^−1^ and ${k_2}/{K_1}$ = 0.05. Each color marker represents the average over 100 noise replicates for three mixing cases: M1 & V = 0.0 (blue), M3 & V = 0.1 (black), and M4 & V = 0.2 (red). All results use the least squares as the fit metric. The error bars represent the standard deviation of the 100 noise replicates. The horizontal offset at each percentage is to distinguish the mixing cases.

In all the plots of figure 4 and for the greater simulation range the physical parameters and mixing-matrix elements we simulate and measure (see supplementary material for figures S.2 and S.3), we observe the uncertainty for each physical parameter (i.e. the standard deviation calculation as represented by the error bar) is linear with the Gaussian noise value. Furthermore, as highlighted by the three cases in figure 4, greater mixing produces a larger uncertainty with a general trend towards a positive bias in the relative error measurements of ${K_1}$ and ${k_2}$ and likewise for the absolute bias measurement of V. However, the uncertainty is such that the measurements are within ≈±1 standard deviation of a 0 relative error or bias. As illustrated in the maximum-mixing case (M4 & V = 0.2), we observe that ${k_2}$ is the most sensitive to noise (≈39% error in measurement at 10% Gaussian noise) followed by the blood-volume fraction (≈30%), and then ${K_1}$(≈10%).

Next, we compared the three different fit metric weighting models into our fitting metric calculation, ${\tilde \chi ^2}$. Figure 5 shows the relative error for ${K_1}$ and ${k_2}$ as well as the absolute bias for V as a function of Gaussian noise for the M3 & V = 0.1 mixing case (the black square markers of figure 4, which used least squares as the fitting metric) using all three weighting models defined in section 2.4. As with figure 4, the horizontal offset at each noise percentage is to differentiate the three weighting models, and we use the same values of ${K_1}$ and ${k_2}$. In all three plots of figure 5 as well as for the greater parameter space, we observed close agreement between the three weighting models. No uncertainty model clearly demonstrates better accuracy (i.e. less bias in the measurements of figure 5) in comparison to the others; all results are well within one standard deviation of each other. Dedicated study of the precision of the results via the estimated uncertainty (see the error bars of figure 5) shows the Gaussian uncertainty weighting was systematically the lowest followed by the Poisson uncertainty weighting with then the uniform weighing of least squares having the greatest uncertainty by comparison. For example, for the mixing case shown in figure 5 a 6% Gaussian noise on the simulated observed concentrations results in an approximate 5% relative error for ${K_1}$ using Gaussian weighting in comparison to about 6% for the least squares weighting model.

**Figure 5.**
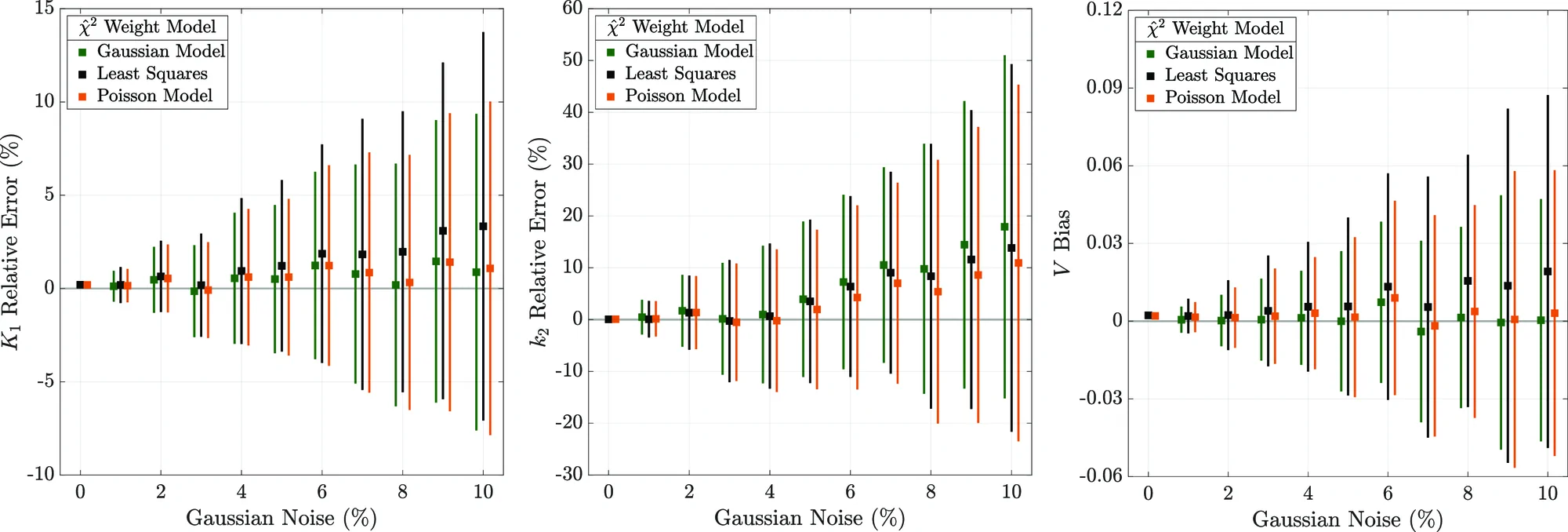
The relative error for ${K_1}$ (left) and ${k_2}$ (middle) as well as the absolute bias for V (right) versus the Gaussian noise percentage on the observed TACs. These results use ${K_1}$ = 1.5 ml min^−1^g^−1^ and ${k_2}/{K_1}$ = 0.05. Each color marker represents the average over 100 noise replicates for three weighting models: Gaussian model (green), least squares or uniform weighting (black), and Poisson model (orange). All results use the M3 & V = 0.1 mixing case. The error bars represent the standard deviation of the 100 noise replicates. The horizontal offset at each percentage is to distinguish the weighting models. There is no Gaussian model data point for 0% noise.

### 3.3. Error propagation estimation

Following the method outlined in section 2.5.3, we investigate the effect of having uncertainty on our mixing-matrix elements using our MC simulation and compare these results to a direct analytical approximation using error propagation estimation via equation (10). We observe that the relative uncertainty (${s_P}/P$) results in an increasing but concave-down trend as the uncertainty of our mixing-matrix elements ($\sigma \left( {{M_i}} \right)$) increases for any combination of mixing-matrix elements using the MC results. By construction, the analytical approximation of the relative uncertainty is linear with $\sigma \left( {{M_i}} \right)$. As an extreme example, when the only uncertainty considered is for ${R_A}$, we find the error in ${K_1}$ to be completely linear and well described by our analytical prediction across the full range of uncertainty on ${R_A}$ and for all mixing matrices considered. More generally, the relative uncertainty (${s_P}/P$) results are approximately linear for low uncertainties of ${M_i}$ ($\sigma \left( {{M_i}} \right) \lesssim $ 0.2) for mixing matrices 3–4 (M3–M4), and therefore, the analytical approximation (${\sigma _P}/P$) well describes this regime. This is also true for ${R_T}$ and ${R_A}$ when we consider mixing matrices 1–2 (M1–M2) but not for ${F_T}$ and ${F_A}$, which deviate from linearity at our first non-zero measurement of $\sigma \left( {{M_i}} \right)$ = 0.01.

We illustrate these results in figure 6 where we consider relative uncertainty for all three physical variables in the case where the uncertainty is uniformly increasing on each mixing-matrix element. The examples shown are for ${K_1}$ = 1.5 ml min^−1^g^−1^, ${k_2}/{K_1}$ = 0.05, and V = 0.1 with all four mixing matrices (M1–M4). The colored markers in figure 6 show the MC results while the red line is the first-order analytical approximation of the error propagation via equation (10). Consistent with our more general findings, we observe that the sensitivity to the uncertainty is greatest for V (e.g., ≈60% at $\sigma \left( {{M_i}} \right)$ = 0.05) and also large for ${k_2}$ (e.g., ≈50% at $\sigma \left( {{M_i}} \right)$ = 0.05) for each mixing matrix. By comparison, figure 6 shows a significantly lower (less sensitive) relative error for ${K_1}$ (e.g., ≈14% at $\sigma \left( {{M_i}} \right)$ = 0.05), and it maintains approximate linearity for more of the mixing matrix uncertainty range. Like the behavior of figure 4, we observe that increasing the mixing matrix from M1 to M4, results in a greater relative uncertainty increase for increased mixing (i.e. greater slope of ${\sigma _P}/P$ with $\sigma \left( {{M_i}} \right)$). The analytical approximation shown in figure 6, is an accurate descriptive of the MC results for M2–M4 but does not describe the observed concave-down non-linearity starting around $\sigma \left( {{M_i}} \right) \gtrsim $ 0.2. For M1, the analytical approximation does not accurately describe the rate of change (slope) of ${\sigma _P}/P$ with $\sigma \left( {{M_i}} \right)$ and therefore greatly overestimates the relative uncertainty given by the MC results.

**Figure 6.**
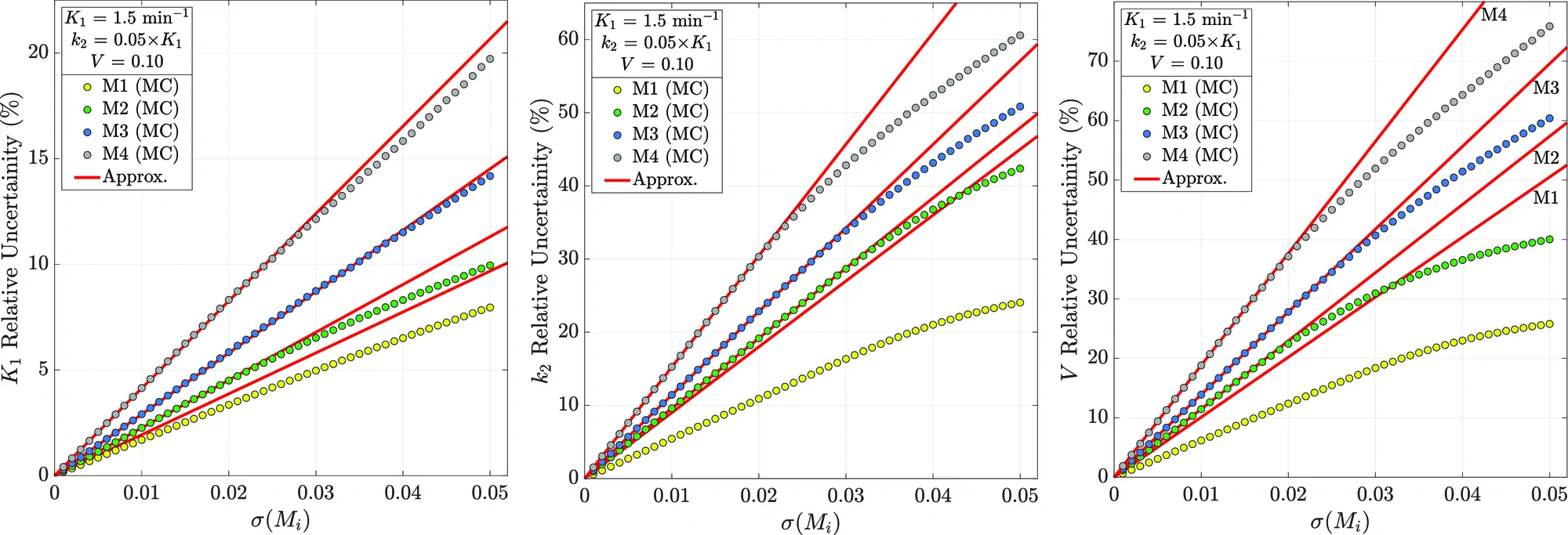
The relative uncertainty versus the Gaussian uncertainty in each of the four mixing-matrix elements, $\sigma \left( {{M_i}} \right)$, where P is *K*_1_ (left), ${k_2}$ (middle), and V (right). The markers represent the MC results (${s_P}/P$) for M1–M4 while the red line is the analytical approximation (${\sigma _P}/P$) denoted as ‘Approx.’ where M1 to M4 corresponds to increasing slope. As annotated in the legend, these results are for ${K_1}$ = 1.5 ml min^−1^g^−1^, ${k_2}/{K_1}$ = 0.05, and V = 0.1.

The analytical approximation of the uncertainty for a given physical parameter using equation (10) assumed no correlation between the randomly generated mixing-matrix elements. Using the MC simulation, we found that this assumption held true when the uncertainty was low. For example, when $\sigma \left( {{M_i}} \right)$ < 2.5 for all elements of the M3 mixing matrix, the correlation between mixing-matrix elements was within ±0.005 and typically with a *p*-value > 0.05. However, we observed that for two mixing-matrix element pairs, ${R_T}$ with ${F_T}$ and ${R_A}$ with $F_A$, there was a positive correlation as $\sigma \left( {{M_i}} \right)$ increased from ≈2.5. For the M3 mixing matrix, the correlation (for both pairs) was approximately 0.07 at $\sigma \left( {{M_i}} \right)$ = 0.05 with a *p*-value < 0.001.

## 4. Discussion

Our in-house fitting software was able to reliability fit the simulated TACs across our prescribed parameter space involving $K_1$, ${k_2}$, V, and the mixing-matrix elements ${M_i}$. This remained true when considering the effect of adding noise (see section 3.2) as well as different fit metrics given by our weighting models.

Figure 1 (right) illustrates a single realization where 3% Gaussian noise applied. Qualitatively we find ≈3%–4% describes the scale of the noise variation we see with mouse data TAC (Guerraty *et al* 2021) using the same 10 s time-frame binning. However, the noise we generate is, by construction, independent frame-to-frame and VOI-to-VOI. In addition to not accounting for systematically correlated effects, our noise generation does not attempt to model aspects like respiratory motion or, more generally, the assumptions that the mixing-matrix elements are spatially and time independent, etc. Nevertheless, these results allow us to evaluate our fitting software in conjunction with the kinetic model (Guerraty *et al* 2021, Johnson *et al* 2023). We have previously investigated (Johnson *et al* 2023) the accuracy and uncertainty of the physical parameters using our fitting software (as was done herein) when changing the input function model or the time-frame binning. These effects are understood. Moreover, the results of this analysis are not contingent on the exact form of the input function and are in the context of a fully analytical understanding of the fit in relation to which parameters are included (‘free’) in the fit versus which are set (‘fixed’) parameters.

### 4.1. Structural identifiability

In section 3.1, we showed that systematically biasing ${F_*}$ (${F_T}$ or ${F_A}$) across the physical range predicted by (Metzler *et al* 2026) results in a bias in the measured physical parameters (figures 2 and 3). However, across this forced biased ${F_*}$ range where the other three mixing-matrix elements are known and used as set parameters, we observed a nearly uniform (flat) fitting metric. Our fits of simulated data thus confirm the results of (Metzler *et al* 2026); ${K_1}$ and V are not structurally identifiable in the ${F_A}$ free (i.e. unknown) fit parameter case (section 3.1.1) and, likewise, ${K_1}$, ${k_2}$, and V are not structurally identifiable in the ${F_T}$ free (i.e. unknown) fit parameter case (section 3.1.2). Outside the forced biased ${F_*}$ range, the fitting metric would rapidly increase. We conclude, consistent with the mathematical derivation in (Metzler *et al* 2026), that we have *local non-identifiability* (Chis *et al* 2011, Guillaume *et al* 2019, Wieland *et al* 2021) in the previously stated cases. However, this is of little value operationally as the true simulated physical parameters will be within the local region (i.e. ${F_*}$ range). This conclusion follows for any reversible one-compartment model (equation (1)) using our kinetic model framework (equation (2)). Therefore, the implications of (Metzler *et al* 2026) demonstrated here, is that all four elements of the mixing matrix need to be estimated (nominally with some estimation of uncertainty) in order to extract the physical parameters from the kinetic fit.

As a contrasting example, in order to achieve identifiability for ${K_1}$ and ${k_2}$ (Ocneanu *et al* 2017) assumed ${R_T} + {F_A}$ = 1 and ${R_A} + {F_T}$ = 1 (ideal quantification of the activity concentrations), and the physiological blood-volume fraction was subsumed into the mixing matrix (effectively V = 0). However, in our exploration, we found the sum of the mixing-matrix rows did not match unity for mice in SPECT (Guerraty *et al* 2021). Additionally, we seek to measure the blood-volume fraction particularly in the context of rest versus stress dynamics (Mohy-Ud-Din *et al* 2018, Yousefi *et al* 2023). Thus, there may be certain tradeoffs (e.g. the distinction between V and ${F_A}$) and justifiable assumptions (e.g. detector performance with respect to the recovery coefficients for measured for the VOIs) that allow for fitting a simplified mixing-matrix configuration (Hutchins *et al* 1992, Klein *et al* 2010, Ocneanu *et al* 2017).

The results of Johnson *et al* (2023) assumed ${k_2}$ = 0 and discovered ${F_T}$ as a free fit parameter would be globally structurally identifiable for ${K_1}$ and V. We confirmed that this held true in the results presented here and for any case where ${k_2}$ was a fixed constant. The conclusion of Johnson *et al* (2023) regarding $F_A$ as a free fit parameter is expanded for all values of ${k_2}$ in addition to the kinetically irreversible case (${k_2}$ = 0). Additionally, ${k_2}$ is identifiable and can be accurately measured (with low bias) with high precision (low variance) in the noiseless data.

Figures 2 and 3 provide an insight into the uncertainty sensitivity of the physical parameters, i.e. how susceptible a change in the bias of the assumed value of the mixing-matrix elements (e.g. ${F_A}$) results in bias in measured parameters. For example, in figure 2 we observe that the relative error in V changes quicker than for ${K_1}$. In figure 3, we found ${k_2}$ to be extremely sensitive to bias followed by ${K_1}$ and then V. Unsurprising, when we applied the error analysis of section 2.5.3, we found the same trend when we considered uncertainty only on ${F_A}$ or ${F_T}$, respectively.

### 4.2. Fitting parameter uncertainty due to noise

In contrast to section 3.1, the results of section 3.2 give an indication on the more open issue of the practical identifiability of the physical variables due to noise. We observed a linear response from the induced Gaussian noise to the statistical uncertainty of all the physical variables via the fitting measurement (figures 4 and 5). Thus, the uncertainty was predictable and consistent across the 0%–10% noise level. This held true across all mixing cases but greater mixing (e.g. M1 & V = 0.0 compared to M4 & V = 0.2) gave a greater uncertainty relative to the Gaussian noise level, as anticipated. These trends held true across the kinetic range of ${K_1}$ and ${k_2}$ explored. We advised caution if applying these results using the same kinetic model but with a different experimental setup (i.e. the tracer properties, detector system, scanning protocol, etc). For example, the precise measurement of ${k_2}$ is governed not only by the noise level but also by the scan duration as well as the temporal resolution (time-frame binning), which is contingent on the system sensitivity.

The least uncertain physical variable was ${K_1}$ (e.g. under 5% uncertainty for 5% noise with M3 & V = 0.1), which is the primary variable of interest for extracting the MBF. As noted before, ≈3%–4% noise is a better description of the noise profile on our activity concentrations that we measure in data. The uncertainties grew faster, by comparison, for $k_2$ and V (≳15% uncertainty in ${k_2}$ for 5% noise with M3 & V = 0.1 as shown in figure 4). Therefore, if we specified a maximum uncertainty tolerance for each of the physical parameters then ${K_1}$ is more practically identifiable for a greater noise level relative to ${k_2}$ and V. Other work (Raue *et al* 2009, Wieland *et al* 2021), allows for setting the tolerance based on statistical significance (i.e. confidence level). This could be achieved in this work by noting that ${\tilde \chi ^2}$ (see equation (3)) becomes a ${\chi ^2}$ estimator for the Gaussian weighting model. However, directly using the uncertainty tolerance on the physical parameter of interest (e.g. ${K_1}$) is more valuable for our purposes. Moreover, with realistic data, an ideal estimator for the uncertainty (noise) on the concentration is not known and this choice of noise model can therefore affect the apparent statistical significance.

The Gaussian weighting model gave the smallest uncertainty (figure 5) in the physical variables but was only nominally better than the Poisson weighting model and never more than ≈40% away from the least squares (uniform weighting) fitting metric that we use by default in our mouse studies (Guerraty *et al* 2021). In this controlled work using simulations, the method of noise generation was artificial and the observed favoring of the fitting metric that utilized that exact model was unsurprising. Only using Gaussian noise was a limitation of this work, but the trends between weighting models were consistent (i.e. for different mixing or kinetic parameters) and proportional, especially between the Gaussian and Poisson weighting.

The results of section 3.2 do show signs of a slight systematic bias as noise increases (see figure 4). However, the statistical uncertainty in the measurement of the physical parameters grows faster than the bias and is therefore consistent with zero bias. The improvement in accuracy (see figure 5) for and V is interesting and does suggest again the potential benefit of incorporating some type of uncertainty-based weighting in future analysis. In combination with the more compelling improvement (reduction) in the uncertainty compared to least-squares weighting, some type of concentration-is-proportional-to-error weighting would be favorable with real noisy data, and the Poisson weighting model provides easy estimation of the activity concentration uncertainty at each time-bin for this purpose. However, this work and investigation of other weighting models like nonlinear least squares are beyond the scope of this paper.

### 4.3. Error propagation estimation

As mentioned in section 4.1, the results of figures 2 and 3 are more instructive when thinking about the potential propagated error (uncertainty) in the measurement of the three physical parameters. Section 3.3 presented results that utilized a MC to estimate the uncertainty on each physical parameters for different levels of absolute uncertainty on the mixing-matrix elements in addition to analytical based error propagation estimation. Like the results of section 3.1 and the discussion of section 4.1 we found that increasing the uncertainty on each of the mixing-matrix elements ($\sigma \left( {{M_i}} \right)$) produced greater uncertainty in ${k_2}$ and V compared to ${K_1}$ (see figure 6). The results of this analysis are that uncertainty or error in ${K_1}$ is the least sensitive to change in uncertainty of the input mixing-matrix elements. As an example, the M3 & V = 0.1 MC results in figure 6 produced an uncertainty ≈ 14% for ${K_1}$ given an absolute error of 0.05 in $\sigma \left( {{M_i}} \right)$ for all four mixing-matrix elements.

As with section 3.2, the effect of changing the mixing matrix correlated to greater relative uncertainty on the physical quantities (compare figures 4–6). As noted in section 4.2, this is consistent with the limitation of the fitting model when there is uncertainty. One caveat with our presentation of figure 6 is that we show results using the absolute error in ${M_i}$ where there is (equal) uncertainty on all four mixing elements. The relative error on each of the parameters is thus very different for the recovery coefficients (${R_*}$) compared to the spillover/mixing-fractions (${F_*}$). To use mixing matrix M2 and $\sigma \left( {{M_i}} \right)$ = 0.01 as an example, the relative error of ${R_T}$ is ≈1.1% while for the relative error of ${F_T}$ is 10.0%. However, this is not limitation of the model; in our use case, we would apply the estimated uncertainty for each mixing-matrix element and then estimate the relative uncertainty in the physical parameters using our MC method.

The example plots shown in figure 6 also highlight that the analytical approximation of propagated uncertainty on the mixing-matrix elements is always an overestimate in comparison to the gold standard of the MC. We caution that for a different kinetic model or parameter space this may not always hold true. Therefore, the analytical approximation is useful when in well understood location in model-parameter space. A full MC treatment avoids the limitation of first order approximation of the uncertainty propagation and provides a greater understanding of the effect at the level of the distribution of the physical events at the cost of computation, which was relatively minor in this analysis. This full treatment was only possible due to the understanding afforded by Metzler *et al* (2026), which enable us to compute the modified physical parameters given the uncertainty of the mixing-matrix elements via equations (4)–(6).

The effect noted in section 3.3 regarding the poor agreement of the analytical approximation for M2 and especially M1 is well understood. This effect comes from the assumption of a normal distribution with standard deviation $\sigma \left( {{M_i}} \right)$ about the true ${M_i}$ value convoluted with our MC event selection (veto) requirements. For M1, the selection for ${F_T}$ and ${F_A}$ has an absolute minimum at 0 (i.e. ${F_*} \unicode{x2A7E} $ 0); the mean of resulting distribution is therefore skewed away from the true value (${F_T}$ = ${F_A}$ = 0). Additionally, the requirement that the physical variables (see equations (4)–(6)) be physically bounded (e.g. $V \unicode{x2A7E} $ 0) can further skew the distribution of ${F_T}$ and ${F_A}$ and to a lesser extent ${R_T}$ and ${R_A}$ due to events being vetoed. Thus, for M1, the resulting distribution for ${F_T}$ and ${F_A}$ are not representative of a normal distribution centered at the true value with a standard deviation given by the set absolute uncertainty $\sigma \left( {{M_i}} \right)$. This effect is still present for M2 (especially for ${F_A}$ = 0.05) when the absolute uncertainty is large. Thus, the constraint that our parameters be physical results in the input distributions for ${M_i}$ potentially not being truly Gaussian and/or not at the prescribed mean and standard deviation. Likewise, the resulting (output) physical distributions of events are skewed to be non-normal distributions. In particular, the leading edge of the distributions for both V and ${k_2}$ (but not ${K_1}$) are truncated at the physical limit ($V \unicode{x2A7E} $ 0 and ${k_2} \unicode{x2A7E} $ 0) due to the MC event selection veto. These effects were predicted in the numerical trials section of (Metzler *et al* 2026) and are just a reflection of the underlining math. As noted, the distribution of events for ${K_1}$ was always positive in the totality of our parameter space.

## 5. Conclusion

Using simulations and our previously incorporated kinetic model and fitting software (Guerraty *et al* 2021, Johnson *et al* 2023), we confirmed the conclusion of Metzler *et al* (2026): the simultaneous structural identifiability the tissue-uptake rate, ${K_1}$, the compartment-washout rate, ${k_2}$, and the blood-volume fraction, V, requires prior knowledge of all mixing-matrix elements in equation (2). In this context, our investigation of the uncertainty on the three physical parameters of the kinetic model led to two general conclusions. First, increasing the noise on the observed activity concentrations leads to an approximate linear increase in uncertainties. Second, the uncertainty of the physical parameters is, likewise, proportional to uncertainty or bias in the mixing-matrix elements.

## Data Availability

The data cannot be made publicly available upon publication because they are not available in a format that is sufficiently accessible or reusable by other researchers. The data that support the findings of this study are available upon reasonable request from the authors. Supplementary Material available at https://doi.org/10.1088/1361-6560/ae62c5/data1.

## Appendix

Given the uncertainty for each mixing-matrix element (${\sigma _i} = \sigma \left( {{M_i}} \right)$) we calculate the uncertainty in each of the physical fit parameters (${\sigma _{{K_1}}} = \sigma \left( {{K_1}} \right)$, ${\sigma _{k2}} = \sigma \left( {{k_2}} \right)$, and ${\sigma _V} = \sigma \left( V \right)$) using equation (10). The uncertainties are shown in equations (A.1)–(A.3) and represent an analytical approximation of the error propagation in contrast to the simulated uncertainty given by the Monte Carlo (see section 2.5.3)

\begin{equation*} {\sigma _{K1}} = \left| {\frac{{{K_1}}}{X}} \right|\sqrt {\mathop {\mathop \sum \nolimits }\limits_{\forall {\text{ }}i} {{\left( {\frac{{\partial X^{\prime}}}{{\partial M_i^{\prime}}}{\Bigg|_M}} \right)}^2}\sigma _i^2} \end{equation*}

\begin{equation*} {\sigma _{{k_2}}} = \left| {\frac{{{K_1}{R_T}{F_T}\left( {1 - V} \right)}}{{{\Gamma }}}} \right|\sqrt {\frac{{\sigma _{{R_T}}^2}}{{R_T^2}} + \frac{{\sigma _{{F_T}}^2}}{{F_T^2}}} \end{equation*}

\begin{equation*} {\sigma _V} = \frac{{\sqrt {{\delta ^2}\left( {{V^2}\sigma _{{R_T}}^2 + \sigma _{{F_A}}^2} \right) + {\beta ^2}\left( {{V^2}\sigma _{{F_T}}^2 + \sigma _{{R_A}}^2} \right)} }}{{\left| {{\Gamma }} \right|}}.\end{equation*}

For equation (A.1), the partial derivatives of $X^{\prime}$ (see equation (8)) are given in equations (A.4)–(A.7) and are evaluated at the true mixing matrix, M, as noted in section 2.5.3 regarding equation (10)

\begin{equation*} {{\left. {\frac{{\partial X^{\prime}}}{{\partial R_T^{\prime}}}} \right|}_M} = + \left( {\frac{{{F_A}}}{{{\beta ^2}}} + \left( {\frac{{R_T^2}}{{{\beta ^2}}} - \frac{{F_T^2}}{{{\delta ^2}}}} \right)\frac{\delta }{{{\Gamma }}}V} \right)\left( {1 - V} \right) + \left( {\frac{{{R_T}}}{\beta } - \frac{{{F_T}}}{\delta }} \right)\frac{\delta }{{{\Gamma }}}V \end{equation*}

\begin{equation*} {{\left. {\frac{{\partial X^{\prime}}}{{\partial R_A^{\prime}}}} \right|}_M} = + \left( {\frac{{{F_T}}}{{{\delta ^2}}} - \left( {\frac{{R_T^2}}{{{\beta ^2}}} - \frac{{F_T^2}}{{{\delta ^2}}}} \right)\frac{\beta }{{{\Gamma }}}} \right)\left( {1 - V} \right) - \left( {\frac{{{R_T}}}{\beta } - \frac{{{F_T}}}{\delta }} \right)\frac{\beta }{{{\Gamma }}} \end{equation*}

\begin{equation*} {{\left. {\frac{{\partial X^{\prime}}}{{\partial F_T^{\prime}}}} \right|}_M} = - \left( {\frac{{{R_A}}}{{{\delta ^2}}} + \left( {\frac{{R_T^2}}{{{\beta ^2}}} - \frac{{F_T^2}}{{{\delta ^2}}}} \right)\frac{\beta }{{{\Gamma }}}V} \right)\left( {1 - V} \right) - \left( {\frac{{{R_T}}}{\beta } - \frac{{{F_T}}}{\delta }} \right)\frac{\beta }{{{\Gamma }}}V \end{equation*}

\begin{equation*} {{\left. {\frac{{\partial X^{\prime}}}{{\partial F_A^{\prime}}}} \right|}_M} = - \left( {\frac{{{R_T}}}{{{\beta ^2}}} - \left( {\frac{{R_T^2}}{{{\beta ^2}}} - \frac{{F_T^2}}{{{\delta ^2}}}} \right)\frac{\delta }{{{\Gamma }}}} \right)\left( {1 - V} \right) + \left( {\frac{{{R_T}}}{\beta } - \frac{{{F_T}}}{\delta }} \right)\frac{\delta }{{{\Gamma }}} .\end{equation*}

The terms β and δ are via (Metzler *et al* 2026) and defined as follows along with the definition of ${{\Gamma }}$, which is the determinant of the mixing matrix

\begin{equation*}\begin{array}{*{20}{c}} {\beta = {R_T}V + {F_A}} \end{array}\end{equation*}

\begin{equation*}\begin{array}{*{20}{c}} {\delta = {R_A} + {F_T}V} \end{array}\end{equation*}

\begin{equation*}\begin{array}{*{20}{c}} {\Gamma = {R_T}{R_A} - {F_T}{F_A}} \end{array}\end{equation*}

## References

[pmbae62c5bib1] Akima H (1970). A new method of interpolation and smooth curve fitting based on local procedures. J. ACM.

[pmbae62c5bib2] Akima H (1974). A method of bivariate interpolation and smooth surface fitting based on local procedures. Commun. ACM.

[pmbae62c5bib3] Boschi A, Uccelli L, Marvelli L, Cittanti C, Giganti M, Martini P (2022). Technetium-99m radiopharmaceuticals for ideal myocardial perfusion imaging: lost and found opportunities. Molecules.

[pmbae62c5bib4] Camici P G, Crea F (2007). Coronary microvascular dysfunction. New Engl. J. Med..

[pmbae62c5bib5] Cherry S R, Sorenson J A, Phelps M E Physics in nuclear medicine.

[pmbae62c5bib6] Chis O T, Banga J R, Balsa-Canto E (2011). Structural identifiability of systems biology models: a critical comparison of methods. PLoS One.

[pmbae62c5bib7] Del Buono M G, Montone R A, Camilli M, Carbone S, Narula J, Lavie C J, Niccoli G, Crea F (2021). Coronary microvascular dysfunction across the spectrum of cardiovascular diseases: JACC state-of-the-art review. J. Am. Coll. Cardiol..

[pmbae62c5bib8] Gábor A, Villaverde A F, Banga J R (2017). Parameter identifiability analysis and visualization in large-scale kinetic models of biosystems. BMC Syst. Biol..

[pmbae62c5bib9] Guerraty M A, Johnson L C, Blankemeyer E, Rader D J, Moore S C, Metzler S D (2021). Development and feasibility of quantitative dynamic cardiac imaging for mice using muSPECT. J. Nucl. Cardiol..

[pmbae62c5bib10] Guerraty M A, Rao H S, Anjan V Y, Szapary H, Mankoff D A, Pryma D A, Rader D J, Dubroff J G (2020). The role of resting myocardial blood flow and myocardial blood flow reserve as a predictor of major adverse cardiovascular outcomes. PLoS One.

[pmbae62c5bib11] Guillaume J H A (2019). Introductory overview of identifiability analysis: a guide to evaluating whether you have the right type of data for your modeling purpose. Environ. Modelling Softw..

[pmbae62c5bib12] Hutchins G D, Caraher J M, Raylman R R (1992). A region of interest strategy for minimizing resolution distortions in quantitative myocardial PET studies. J. Nucl. Med..

[pmbae62c5bib13] Johnson L C, Guerraty M A, Moore S C, Metzler S D (2019). Quantification of myocardial uptake rate constants in dynamic small-animal SPECT using a cardiac phantom. Phys. Med. Biol..

[pmbae62c5bib14] Johnson L C, Guerraty M A, Moore S C, Metzler S D (2023). Determining the effect of cardiac blood volume on accuracy of uptake rate constants by simulation. Phys. Med. Biol..

[pmbae62c5bib15] Klein R, Beanlands R S, deKemp R A (2010). Quantification of myocardial blood flow and flow reserve: technical aspects. J. Nucl. Cardiol..

[pmbae62c5bib16] Metzler S D, Stentz D J, Guerraty M A (2026). Simultaneously quantifying K_1_, k_2_, and blood volume in myocardial imaging with overlapping volumes of interest: structural identifiability. Phys. Med. Biol..

[pmbae62c5bib17] Mohy-Ud-Din H, Boutagy N E, Stendahl J C, Zhuang Z W, Sinusas A J, Liu C (2018). Quantification of intramyocardial blood volume with (99m)Tc-RBC SPECT-CT imaging: a preclinical study. J. Nucl. Cardiol..

[pmbae62c5bib18] Ocneanu A F, deKemp R A, Renaud J M, Adler A, Beanlands R S B, Klein R (2017). Optimally repeatable Kinetic model variant for Myocardial blood flow measurements with (82)Rb PET. Comput. Math. Methods Med..

[pmbae62c5bib19] Raue A, Kreutz C, Maiwald T, Bachmann J, Schilling M, Klingmuller U, Timmer J (2009). Structural and practical identifiability analysis of partially observed dynamical models by exploiting the profile likelihood. Bioinformatics.

[pmbae62c5bib20] Scarica V, Rinaldi R, Animati F M, Manzato M, Montone R A (2025). Coronary microvascular dysfunction: pathophysiology, diagnosis, and therapeutic strategies across cardiovascular diseases. EXCLI J..

[pmbae62c5bib21] Schindler T H (2023). Myocardial perfusion PET for the detection and reporting of coronary microvascular dysfunction: a JACC: cardiovascular imaging expert panel statement. JACC Cardiovasc. Imaging.

[pmbae62c5bib22] Wieland F-G, Hauber A L, Rosenblatt M, Tönsing C, Timmer J (2021). On structural and practical identifiability. Curr. Opin. Syst. Biol..

[pmbae62c5bib23] Yousefi H (2023). Quantification of intramyocardial blood volume using (99m)Tc-RBC SPECT/CT: a pilot human study. J. Nucl. Cardiol..

[pmbae62c5bib24] Zuo Y, Sarkar S, Corwin M T, Olson K, Badawi R D, Wang G (2019). Structural and practical identifiability of dual-input kinetic modeling in dynamic PET of liver inflammation. Phys. Med. Biol..

